# Effects of a Scapular-Focused Exercise Protocol for Patients with Rotator Cuff-Related Pain Syndrome—A Randomized Clinical Trial

**DOI:** 10.3390/jfmk10040475

**Published:** 2025-12-09

**Authors:** Cristina dos Santos, Isabel Bastos de Almeida, Mark A. Jones, Ricardo Matias

**Affiliations:** 1 Physiotherapy Department, Superior School of Health of Alcoitão, Rua Conde Barão, 2649-506 Alcabideche, Portugal; 2 Physiotherapy Department, Superior School of Health, Polytechnic Institute of Setúbal, 2910-761 Setúbal, Portugal; isabel.bastos@ess.ips.pt; 3 Allied Health and Human Performance, Adelaide University, Adelaide, SA 5005, Australia; jonesma56@gmail.com; 4 Kinetikos, 3030-199 Coimbra, Portugal; rmatias@kinetikoshealth.com; 5 Physics Department & Institute of Biophysics and Biomedical Engineering (IBEB), Faculty of Sciences, University of Lisbon, 1749-016 Lisbon, Portugal

**Keywords:** rotator cuff injuries, shoulder pain, activities of daily living, scapula, electromyography, feedback

## Abstract

**Background**: Current clinical practice still lacks consistent evidence in the physiotherapy management of rotator cuff-related pain syndrome (RCS). The purpose of this trial was to compare the effectiveness of a scapular-focused treatment with and without real-time electromyographic biofeedback (EMGBF) to a control therapy in patients with RCS, in the short-term. **Methods**: 60 patients with RCS were divided into three groups: the scapular-focused exercise protocol group (P_G *n* = 20), the scapular-focused exercise protocol with EMGBF group (P+EMGBF_G *n* = 20), and the control therapy group (CT_G *n* = 20). Values of pain and function [Shoulder Pain and Disability Index (SPADI) questionnaire, complemented by the Numeric Pain Rating Scale (NPRS) and Disabilities of the Arm, Shoulder and Hand (DASH) questionnaire], scapular stabilizer neuromuscular control (SSNC), scapular stabilizer activation onset (SSAO), dynamic scapular alignment, range of motion (ROM), and glenohumeral flexor and abductor muscle strength (GMS) were assessed at baseline and after 6 weeks and compared within and between groups. **Results**: There were significant differences in pain and function, SSNC, SSAO, dynamic scapular alignment, ROM, and GMS in all groups between the initial and 6-week assessments. However, the P+EMGBF_G showed superior results in pain and function, SSNC, and dynamic scapular alignment than the CT_G and superior results in SSNC than the P_G. The P_G had superior results in pain and function and dynamic scapular alignment than the CT_G. **Conclusions**: This trial supports the use of a scapular-focused exercise protocol as a comparative approach that effectively improves pain and function in patients with rotator cuff-related shoulder pain syndrome. These results in pain and function were shown to be independent of the use of EMGBF.

## 1. Introduction

Rotator cuff disorders are the most frequent group of pathologies affecting the shoulder and represent 50–85% of shoulder conditions treated by health professionals [1]. Rotator cuff-related pain syndrome (RCS) is commonly reported in the general population, and a common cause of disability at work and during daily activities [2,3,4,5]. Shoulder pain can therefore be seen as an important medical and socioeconomic problem in Western society [1].

RCS is characterized by the presence of pain [6,7,8,9], decreased function [5,6,7,8,9], muscle weakness [5,7,8,10,11], altered range of motion (ROM) [5,6,7,9], altered scapula neuromuscular control [7,12], and scapular dyskinesis [8,13,14].

Over time, a variety of physiotherapeutic treatment modalities have been suggested for the treatment of shoulder impingement pain including electrotherapy, exercise therapy, massage, joint mobilization, shockwave treatment, ultrasound or laser treatment, and sling exercise therapy [1,15,16]. Several systematic reviews have evaluated the effectiveness of these modalities in shoulder disorders [15,16,17,18,19,20], however there is limited evidence to support the efficacy of most of these therapeutic interventions on RCS [15,19,21].

The current evidence [1,4,19,22] states that exercise has a statistically and clinically important effect on pain reduction and improved function. In addition, manual therapy seems to augment the effects of exercise, while several studies reported no evidence for the effectiveness of a scapular-focused treatment approach in patients with RCS [16,19,23]. However, other researchers investigating scapular-focused stabilization and motor control exercises to address scapular dyskinesis, reduction in pain [7,8,10,24], and restoration of function [7,8,10], in RCS found it beneficial.

Moreover, dos Santos et al. [7] have suggested that a well-described scapular-focused exercise protocol, with the aid of real-time electromyographic biofeedback (EMGBF) and home-based management, can reduce pain and increase function, as well as scapular neuromuscular activity and control, ROM, and glenohumeral flexor and abductor muscle strength (GMS) in patients with shoulder dysfunctions in the short-term. The long-term results also appear to maintain gains in pain and function, as well as scapular stabilizer activation onset (SSAO), ROM, and GMS gains, but not for scapular stabilizer neuromuscular control (SSNC) or dynamic scapular alignment.

With conflicting research evidence, interventions focused on the scapula should therefore still be considered in treating patients with RCS. However, the effect of adding scapular stabilization exercises to protocols for the progressive strengthening of shoulder complex muscles on a non-multimodal approach remains unclear [1,19].

To advance our understanding of the value of scapular-focused exercise for RCS, a trial was designed with the main objective of comparing pain and function outcomes between three different treatment protocols for patients with RCS, in the short term (6 weeks):

(1) Scapular-focused exercise protocol without EMGBF (P_G);
(2) Scapular-focused exercise protocol supported by real-time EMGBF (P+EMGBF_G);
(3) Control therapy group (CT_G) with manual therapy (glenohumeral joint physiologic and accessory mobilization), massage to reduce upper trapezius (UT) stiffness, and shoulder rotation strengthening into external rotation.

Two hypotheses were put forward:

1. The scapular-focused exercise protocol would produce clinically and statistically superior outcomes over the control therapy.
2. The scapular-focused exercise protocol with EMGBF would produce clinically and statistically superior outcomes over the control therapy.

## 2. Materials and Methods

### 2.1. Study Design

**Figure 1:**
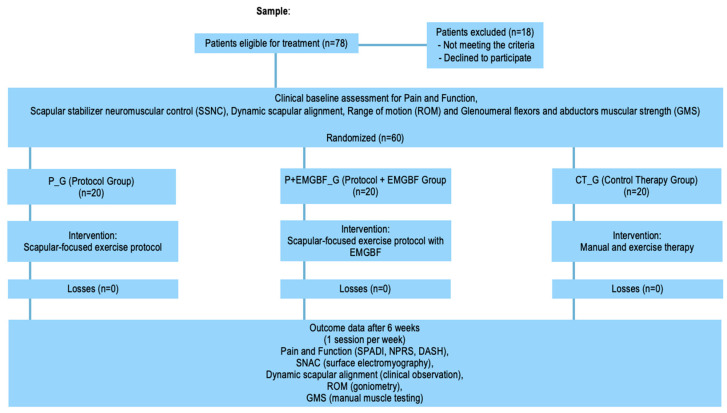
Flowchart illustrating subject enrollment, treatment allocation, and outcome measurements.

A prospective randomized controlled trial was conducted to compare the outcomes of a scapular-focused exercise protocol [7] with and without real-time EMGBF, and a control therapy group receiving shoulder mobilization, UT massage and general upper limb strengthening, with baseline (initial) and 6-week (final) assessments. The flowchart illustrating subject enrollment, treatment allocation and outcome measurements is summarized in Figure 1. The ethics committee of Alcoitão Superior School of Health gave its approval to this trial (CE-ESSAlcoitão, Approval No. 18/2022) and the study has been registered on ClinicalTrials.gov (ID: NCT05516186, Release Date: 23 August 2022).

### 2.2. Participants

Participants were recruited through orthopedic physicians and physiotherapists, working in private medical clinics. Diagnosis was based on the presence of current anterolateral acromial area pain [25], pain with active shoulder elevation [26], pain with passive or isometric resisted shoulder external rotation [27,28], and at least two positive results from the Neer test [29], Hawkins test [30], and Jobe/Empty can test [31].

Participants were included based on the following criteria: 1—age between 18 and 60 years; 2—primary complaint of shoulder pain; and 3—RCS clinical diagnosis. The exclusion criteria were: 1—neurological symptoms [32]; 2—positive thoracic outlet syndrome (screened with Allen’s and Adson’s tests) [32,33]; 3—history of shoulder surgery or fracture [26]; 4—structural injuries confirmed by imaging (e.g., ligaments and labrum); 5—symptoms reproduced by cervical examination [26,34]; 6—unable to commit to scheduled treatments; and 7—anti-inflammatory drug use. These inclusion and exclusion criteria were extracted from dos Santos et al.’s [7] scapular-focused exercise protocol. All eligible participants gave written informed consent before data collection, and they were free to renounce from the study at any moment.

The sample size required for a significance level of 0.05 and a power of 0.80 to detect a significant decrease on the NPRS was calculated to be 363 subjects with RCS. However, the power analysis after 20 treated subjects revealed sufficient power (>0.80) for limiting the subject inclusion to 60.

### 2.3. Randomization and Allocation

From 78 participants recruited, 60 were included and 18 were excluded before starting treatment. These 60 participants were assigned into three groups: P_G, P+EMGBF_G, and CT_G. Each patient was allocated into their group through a computer-generated block randomization process designed to ensure equal sample sizes across groups [35]. The randomization list was prepared by an independent researcher not involved in the assessments or interventions, using random blocks generated in Microsoft Excel. Allocation concealment was maintained by storing the group assignments in sealed opaque envelopes that were opened sequentially and only after each participant had completed baseline assessments. Interventions were delivered by different physiotherapists, each specifically trained and familiarized with the scapular-stability protocol to ensure procedural consistency. Participants were blinded to their group allocation; however, due to the nature of the intervention, therapist blinding was not feasible. Outcome assessors were not blinded, which may introduce a potential risk of performance and detection bias.

All participants’ characteristics are described in Table 1.

### 2.4. Interventions

#### 2.4.1. P+EMGBF_G

The scapular-focused exercise protocol followed the protocol described by dos Santos et al. [7]. The protocol uses the sequential stages of motor relearning (cognitive, associative, and autonomous) as a framework, while promoting the integration of local and global muscle function in weekly sessions divided into three phases. The main purpose of the protocol is to increase scapular neuromuscular activity and control [7].

The real-time EMGBF utilized was the Physioplux system version 1.06. The EMGBF system comprised four pairs of 24 mm-diameter silver chloride gel surface electrodes, a ground electrode of the same type, four electrode pair cables connected to miniaturized differential amplifiers, and a main HUB unit that communicates via Bluetooth™ to a computer. The EMGBF enabled both patients and the physiotherapist to assess, monitor, and correct in real-time the muscular activation and behavior during the exercises. Before placement of the electrodes, patients’ skin was shaved (if necessary) and cleaned with alcohol to reduce skin impedance. Their placement, normalization of electromyographic (EMG) data, and muscle testing positions were the same as those used by dos Santos et al. [7] based on Ekstrom et al. [36] and Hermens et al. [37] (Table 2).

The scapular-focused exercises used for this study and described in Table 3 were based on and inspired by dos Santos et al.’s [7] scapular-focused exercise protocol.

The progression rules (Table 4) followed the dos Santos et al. [7] exercise protocol, where the stimulus magnitude and progression (either in the same exercise or to progress to the next exercise or phase) were tailored to each patient’s performance and re-assessment, while operating within the protocol’s structure. The progression evolved: (1) exercise complexity, (2) feedback from the EMGBF and from the physiotherapist, (3) perceived effort, (4) sets, repetitions, and endurance, and (5) resting time between exercises.

#### 2.4.2. P_G

The same protocol described above was applied without EMGBF. While the EMGBF was not provided, participants did receive external feedback from the physiotherapist through verbal feedback (verbal commands), manual feedback (manual touching for correcting or maintaining the right position), and visual feedback from a mirror.

#### 2.4.3. CT_G

The control therapy group underwent conservative physical therapy, which included both manual and exercise therapy [16]. Manual therapy consisted of 10 min massage of the shoulder region, with the main goal of relaxing the UT, and 20 min of shoulder mobilization. This mobilization included both accessory and physiological mobilizations of the glenohumeral joint. Longitudinal-caudal and post-anterior accessory mobilizations of the humeral head were performed at a grade (i.e., strength) the therapist judged appropriate for the severity and irritability (i.e., ease of aggravation and time for symptoms to settle based on the description provided in the subjective examination), with the aim of reducing symptoms. Flexion, abduction, and rotation (internal and external) physiological mobilizations were similarly performed based on the therapist’s judgment of the severity, irritability, and extent of each movement impairment with the aim of improving ROM. Exercise therapy comprised internal and external rotation strengthening performed with an elastic band (Thera-Band, Akron, OH, USA), each for 20 min. Participants had to perform three sets of 15 repetitions of internal and external rotation (i.e., a total of six sets), standing with the humerus against the trunk (i.e., neutral position), and with 90° elbow flexion without pain or clear fatigue. All exercises were performed respecting the participant’s pain threshold and under the physiotherapist’s supervision who provided verbal feedback to ensure the correct movement pattern was maintained during the exercise. Between each set of exercises, the participant rested for 2 min. The session ended with UT stretching (2 times for 30 s) and instruction for home exercises. For home exercises, elastic bands (Thera-Band, USA) were given with the instruction to perform the same exercises performed during the face-to-face sessions once a day at a time that was convenient.

In terms of exercise progression, ROM, load, repetitions, and series could be increased as well as resistance. When the patient could perform the repetitions of an exercise without difficulty, the load was progressed to the next stronger color theraband. When the patient ROM improved, the exercise position was also progressed into greater elevation at a load they could manage (i.e., a lighter color theraband if necessary).

Assessments and interventions were performed by three physiotherapists, with two of them specifically trained to use the protocol, with and without EMGBF. All outcomes were assessed prior to the beginning of treatment hereinafter referred to as baseline (initial) and at 6 weeks (final) assessments, respectively (Figure 1). The assessment of pain and function were questionnaires, and the other outcomes were assessed using objective measures, which did not require subjective measurement, with the exception of scapular alignment, where observation was used to assess this outcome. The intervention for each group had an hour mean duration, once a week, for 6 weeks (7 sessions).

### 2.5. Outcomes Measures

The primary outcome measure of pain and function was the SPADI, complemented by the NRPS and DASH. The secondary outcome measures of scapular neuromuscular activity and control were a combination of:

• Surface electromyography for the P+EMGBF_G (allowing both patients and physiotherapists to assess, monitor, and correct in real-time the muscular activation and behavior during the exercises);
• Clinical observation of the scapula’s medial and inferior borders to detect scapular dyskinesis (classified as present if one or both medial and inferior borders were observed during the glenohumeral movement or classified as absent if no prominence was observed [12]);
• Range of motion (ROM) measured using a standard plastic goniometer (following the procedures for the glenohumeral joint motion measurements [41] recognizing the limitation of measurement without stabilization [42]);
• Graded glenohumeral flexors’ and abductors’ isometric muscle strength (GMS) measured through isometric manual muscle testing (acknowledging the reduced sensitivity compared to dynamometry [42]). Outcome measures are presented in Table 5.

### 2.6. Statistical Analysis

The statistical analysis was focused on detecting between- and within-group treatment effects (with effect sizes and 95% CIs) at the baseline and 6 weeks (short-term). Data analysis was performed using SPSS software version 1.0.0.1406. The paired-*t* test was used to compare the pain and function, SSNC, scapular alignment, ROM, and GMS in the same group between the pre- and post-treatment assessments. Independent *t* tests were used to test the primary outcome pain and function for differences between the groups. McNemar and chi-square tests were used to test the secondary outcomes (scapular neuromuscular activity and control, ROM and GMS). The significance level was set at 0.05. Although a two-way repeated-measures ANOVA could provide a more comprehensive analysis of group × time interactions, *t*-tests were chosen due to the exploratory nature and limited sample size of this short-term study. This limitation was considered in the interpretation of the findings.

## 3. Results

### 3.1. Global Initial and Final Results

At baseline, all three groups had poor levels of pain and function (shoulder pain and disability index—SPADI, numeric pain rating scale—NPRS, and disabilities of the arm, shoulder, and hand—DASH), decreased scapular neuromuscular activity and control (SSNC, SSAO, and scapular alignment), and decreased ROM and GMS, with no significant differences between groups (*p* > 0.05) (Table 6 and Table 7).

After 6 weeks of intervention, all outcomes improved significantly compared with the baseline (*p* < 0.05). Pain and function met the Minimal Clinically Important Difference (MCID) criteria.

### 3.2. Within Groups (Comparing Results Between the Initial and Final Assessments)

Significant differences were found in primary outcome pain and function (SPADI, NPRS, and DASH) and in secondary outcomes (SSNC, SSAO, fynamic scapular alignment, ROM, and GMS) for both the P_G and P+EMGBF_G. The CT_G also had significant differences, with exception of the secondary outcomes SSNC and dynamic scapular alignment (*p*^a^ < 0.05) (Table 6 and Table 7).

### 3.3. Between Groups (Comparing Results Between Final Assessments)

#### 3.3.1. P_G vs. P+EMGBF_G

No differences were found between the final results of the two groups, except for SSNC, with better results found in the P+EMGBF_G (*p*^b^ < 0.05) (Table 6 and Table 7).

#### 3.3.2. P_G vs. CT_G

There were significant differences between both P_G and CT_G for the pain and function (SPADI, NPRS, and DASH) outcome and for dynamic scapular alignment, in favor of the scapular-focused exercise protocol (*p*^c^ < 0.05) (Table 6 and Table 7).

#### 3.3.3. P+EMGBF_G vs. CT_G

The two groups showed significant differences in the primary outcome pain and function (SPADI, NPRS and DASH) and in the secondary outcomes SSNC and dynamic scapular alignment (*p*^d^ < 0.05) (Table 6 and Table 7) in favor of P+EMGBF_G.

## 4. Discussion

In this trial, the results of 6 weeks (7 sessions) of the scapular-focused exercise protocol for patients with shoulder dysfunction [7] were compared to the results of 6 weeks (7 sessions) of the same protocol with EMGBF and results from 6 weeks (7 sessions) of a control therapy.

The results of this trial support other research [4,7,8,24,27,34,50,51,52,53,54] that a progressive scapular-focused approach incorporating feedback and home management can significantly improve pain and function in RCS.

No differences were found in the initial assessments of participants’ characteristics between groups or in outcomes at baseline, demonstrating the homogeneity of participants in all groups on these factors.

At the end of the trial, each group had significant differences in their results comparing baseline with the final assessment. Analyzed separately, each intervention was successful in improving the primary outcome, pain and function, and the MCID value met in each group. The same results were found for the secondary outcomes, scapular neuromuscular activity and control, ROM, and GMS. Apparently, all interventions are effective when judged on these measures. However, when analyzed separately, some differences could be found.

In their systematic review, Pieters et al. [19] highlighted that the evidence for exercise as an intervention for shoulder subacromial pain is increasing. They claim that exercise therapy should be considered as a main intervention in the management of people with RCS and that manual therapy provides further benefit if used in addition to exercise therapy [19]. This was the treatment combination used in the CT_G of this trial. In their systematic review, Ravichandran et al. [55] concluded that for the best clinical outcome for RCS, scapular-focused exercises should be used, either exclusively or as a part of a shoulder program. A critique of recent literature is that optimal type, frequency, dose, load, sets, repetitions, interval or rest time, and speed remain unclear in most studies [1,9]. The specificity in describing these variables (for both P_G and P+EMGBF_G) is a strength of this trial.

### 4.1. Primary Outcome Pain and Function

Within group analysis, comparing results between the initial and final assessments showed that clinically meaningful changes were achieved for pain and function over time in all three groups with similar and even better results than previous studies incorporating comparable interventions [6,7,8,24,54,56,57,58,59]. The MCID values were met for all three groups; however, despite the good results achieved in each group, significant differences were found between them. The P+EMGBF_G had statistically significant better results over the other two groups, and the P_G had statistically significant better results over the CT_G. The better results found in the two groups that used the scapula-focused exercise protocol corroborates the literature that suggests rehabilitation that incorporates scapular-focused motor control exercises is effective for reducing pain and disability for patients with RCS [6,7,8,19,24,55]. However, it does not corroborate the results of literature that claim better effects in pain reduction with the combination of both manual therapy and shoulder rotation strengthening exercises [9,16,19,20].

### 4.2. Secondary Outcome Scapular Neuromuscular Activity and Control, ROM, and GMS

The initial SSNC results of decreased activity in lower trapezius (LT) and serratus anterior (SA) muscles support the presupposition that shoulder dysfunction is associated with impaired scapulothoracic stabilizer function [13], consistent with the findings in other studies [7,57]. In contrast, Larsen et al. [60] reported a non-significant tendency to a higher level of mean UT, LT, and SA muscle activity in RCS patients compared to those without RCS. As dos Santos et al. [7] highlighted in their study, these findings support that the diagnostic categorization does not predict muscle function; rather, it is the presence of muscle dysfunction that represents either a risk variable that may contribute to pain and disability, or a central nervous system response to pain and threat [61]. The initial SSNC results may reflect dysfunction in the feedforward processing present [39] even before the onset of movement. This general initial motor plan is expected to be fine-tuned using real-time internal feedback mechanisms [7]. Research has shown that with a planning-control model underpinning the assessment and management of motor control/function, conscious movement can be trained with feedback [61], promoting immediate effects on motor strategies [62], and can restore the force-couple activation [63] in the scapular muscles (stabilizers) [7], consistent with the improvement in LT and SA activity in both P_G and P+EMGBF_G of this trial. The superior results obtained in favor of the P+EMGBF_G may suggest that the EMGBF has an important role in the improvement of this outcome.

Concerning SSAO, the initial and final results of this trial corroborate the findings of dos Santos et al. [7], who highlighted that the pattern of activation alone is not responsible for patients’ symptoms and disability, and it can only be considered a potential predisposing factor that may contribute to some patients’ disabilities depending on their individual lifestyle behaviors and requirements. The non-significant differences found in the 3 groups can lead to an interesting question: How important is it to assess SSAO, if it apparently does not distinguish the presence of dysfunction?

The literature challenges the relationship between scapular alignment and RCS, however in this trial, the use of a scapula-focused exercise protocol (with or without EMGBF) resulted in superior results to the control group that did not incorporate scapular-focused exercise. Comparing both scapula-focused approaches, the results were significantly better for the dynamic scapular alignment outcome, in favor of the P+EMGBF_G, suggesting that the use of EMGBF as an aid to the intervention adds to the improvement of a shoulder strengthening program. However, a kinematics analysis would provide more objective data about this outcome as its data include the displacement and orientation of body segments, joint angles, and spatio-temporal gait parameters [64].

While ROM and GMS had statistically significant improvements in all three groups over 6 weeks, no group was better than the others at improving these outcomes. The GMS finding may be attributed to all three groups receiving rotation strengthening.

The first hypothesis that P_G would produce clinically and statistically superior outcomes over the control therapy was confirmed for the primary outcome, pain and function, and for dynamic scapular alignment. However, no differences were found for the secondary outcomes SSAO, SSNC, ROM, and GMS.

The second hypothesis that P+EMGBF_G would produce clinically and statistically superior outcomes over CT_G was also confirmed for the primary outcome, pain and function, and for the SSNC and dynamic scapular alignment outcomes. However, no differences were found for SSAO, ROM, and GMS outcomes.

### 4.3. Trial Limitations

Although participants were blinded to group allocation, the physiotherapists delivering the interventions and the outcome assessors were not. This lack of blinding may have introduced potential performance and detection bias, which should be considered when interpreting the results. Although the post hoc power analysis indicated sufficient statistical power with the included sample, the smaller number of participants compared with the initial estimation may limit the reliability and generalisability of the between-group comparisons. While the superior results for both P_G and P+EMGBF_G over CT_G in pain and function (SPADI, NPRS and DASH) and in SSNC for P+EMGBF_G are sufficient to support the inclusion of scapular-focused exercise for RCS, the limitations of standard goniometry and manual muscle testing to measure ROM and GMS, respectively, require the improvements in those outcomes are interpreted cautiously. Similarly, the superior outcome in dynamic scapular alignment for both P_G and P+EMGBF_G over CT_G should be interpreted cautiously given the subjective assessment used for this outcome. The judgment regarding scapular alignment was made easier by providing explicit criteria for classifying the alignment. Nevertheless, a more objective measure was available using instruments such as the Flock of birds^®^ (Ascension Technology Corporation, Burligton, VT, USA) or even apps with cameras for the measurement, would be more accurate [42]. Lastly, practical limitations did not allow for blinding of the outcome measurement. This would have had less effect on the pain and function primary outcome measures (SPADI, NPRS, and DASH), or the secondary objective SSNC measure, but could have influenced ROM, GMS, and scapular alignment measures. Further studies are needed to assess the effectiveness of a scapular-focused protocol against other rehabilitation approaches and possibly examine the value of providing kinematic feedback [65,66]. Research investigating whether clinical assessments, such as those from Lewis [33], can predict which patients will benefit from the inclusion of scapular-focused exercise, may also assist in identifying the clinical relevance of scapular impairments, such as SSNC and dynamic scapular alignment. Lastly, the present study did not evaluate the medium- or long-term effects of the intervention, which limits the generalizability of the findings over time and should be considered when interpreting the results. That said, a follow-up would be helpful to compare the results in short or long-term.

### 4.4. Trial Strengths and Comparisons with Previous Research

This RCT is distinctive because it compares the effect of a scapular-focused exercise protocol with and without EMBGF, including a control group therapy, increasing the validity of its results. Concerning the scapular-focused protocol, this clinical trial achieved similar results as previous studies reporting the overall benefits of rehabilitation programs for RCS. We believe this study adds to the body of evidence physiotherapists can draw on when developing shoulder rehabilitation for patients with RCS.

## 5. Conclusions

This trial showed that a scapular-focused exercise treatment approach is more effective, and hence superior, for improving pain and function when compared to a control of manual therapy and shoulder rotation strengthening. This superior result in pain and function was independent of the use of EMGBF. However, SSNC and dynamic scapular alignment improvements were more significant using EMGBF as a complement of the scapular-focused protocol. The results of this clinical trial also showed that the secondary outcomes SSAO, ROM, and GMS improved in all three groups with no differences between interventions.

## Figures and Tables

**Table 1:** Participants’ characteristics.

			P_G	P+EMGBF_G	CT_G
Age (mean (sd))			42.2 ± 10.6	40.9 ± 10.1	41.7 ± 10.8
Sex (%)		Women	11 (55.0)	12 (60.0)	8 (40.0)
		Men	9 (45.0)	8 (40.0)	12 (60.0)
Body height (cm) (mean ± sd)			169.9 ± 7.4	170.5 ± 8.1	170.8 ± 7.9
Body weight (kg) (mean ± sd)			67.8 ± 10.0	68.8 ± 10.8	69.7 ± 10.5
Length of symptoms (%)	Acute (0–2 weeks)		1 (5.0)	1 (5.0)	2 (10.0)
	Sub-acute (2–6 weeks)		4 (20.0)	3 (15.0)	3 (15.0)
	Chronic (+6 weeks)		15 (75.0)	16 (80.0)	15 (75.0)
Symptomatic side (%)	Dominant		17 (85.0)	16 (80.0)	16 (80.0)
	Non-Dominant		3 (15.0)	4 (20.0)	4 (20.0)

Abbreviations: EMGBF electromyographic biofeedback, P_G Scapular-focused-exercise protocol group, P+EMGBF_G Scapular-focused exercise protocol + EMGBF group, CT_G Control therapy group, sd standard deviation, cm centimeter, kg kilogram.

**Table 2:** Placement of the electrodes [7].

Muscle	Placement of the Electrodes	Position	Normalization—Muscular Action to Measure Maximum Voluntary Isometric Contraction
Upper Trapezius	Between the C7 spinous process and the lateral tip of the acromion.	Sitting position with no back support. Shoulder abducted to 90° (no abduction in case of pain) with the neck side-bent to the same side, rotated to the opposite side	Pressure applied posteriorly to the head and above the shoulder. Resist head extension and shoulder elevation.
Lower Trapezius	At 2/3 on the line from the root of the spine of the scapula to the 8th thoracic vertebra	Sitting position with no back support. Arm raised above the head in line with the lower trapezius muscle	Pressure applied to the distal third of the arm, opposing arm elevation
Serratus Anterior	Vertically along the mid-axillary line at the 6th rib through the 8th rib	Sitting position with no back support. Shoulder abducted to 125° in the scapular plane.	Pressure applied above the elbow and at the inferior angle of the scapula attempting to de-rotate the scapula
Anterior Deltoid	At one finger width distal and anterior to the acromion	Sitting position with no back support. Place the humerus in slight external rotation to increase the effect of gravity on the anterior fibers.	Pressure applied on the antero-medial surface of the arm, against abduction (ABD) and flexion

**Table 3:** Scapular-focused exercise descriptions.

Exercise Name	Exercise Description
Exercise 1 “V scapular”	In a sitting position, the patient was encouraged to go to the neutral zone of both scapulae, and then asked to bring the scapulae in and down, as if intending to draw a V on their back with both scapulae (“V scapular”).
Exercise 2 Hand row “V scapular”	In a sitting position, with the upper limb relaxed near the trunk, the patient was asked to do the “V scapular”, and then think of the upper limb as a paddle and slowly try to isometrically pull the bed/chair backward.
Exercise 3 Prone “V scapular” palm up	Laying down in the prone position with the hands relaxed along the trunk (0° ABD of the shoulder) with the palm turned up, the patient was asked to do the “V scapular” without lifting the hand. The movement had to be performed only by the scapulae.
Exercise 4 Prone “V scapular” palm down	Laying down in the prone position with the hands relaxed along the trunk (0° ABD of the shoulder) with the palm turned down, the patient was asked to do the “V scapular” without lifting the hand. The movement had to be performed only by the scapulae.
Exercise 5 Airplane “V scapular”	Laying down in the prone position with 45° ABD of the shoulder, elbow extended, and the hands relaxed and facing up, the patient was asked to do the “V scapular” without lifting the hand. The movement had to be performed only by the scapulae.
Exercise 6 T “V scapular”	Laying down in the prone position with 90° ABD of the shoulder, elbow extended, and the hands relaxed and facing down, the patient was asked to do the “V scapular” without lifting the hand. The movement had to be performed only by the scapulae.
Exercise 7 W “V scapular”	Laying down in the prone position with 45° ABD of the shoulder and 90° F of the elbow with the hand relaxed and facing down, the patient was asked to do the “V scapular” without lifting the hand. The movement had to be performed only by the scapulae.
Exercise 8 Beach “V scapular” lifting hands	Laying down in the prone position with 110° ABD of the shoulder and 90° F of the elbow with the hands relaxed and facing down, the patient was asked to do the “V scapular” and then lift the hands without losing the correct position of the scapulae.
Exercise 9 “V scapular” + EXT ROT	In a sitting position with the elbows near the trunk and flexed at 90°, the patient was asked to do the “V scapular” and then extend the elastic band into external rotation with the hands turned up if possible. In case of pain, the hands were facing down. This exercise started with isometric work and progressed to isotonic work.
Exercise 10 “V scapular” + HOR ABD	In a sitting position with the arms elevated at 90° F of the shoulder in the sagittal plane, the elbows almost fully extended (5° F, just to avoid the closed pack position), and the hands at the shoulder level, the patient was asked to do the “V scapular” and then extend the elastic band into horizontal abduction with the hands facing up if possible. In case of pain, the hands were facing down. This exercise started with isometric work and progressed to isotonic work.
Exercise 11 Rambo “V scapular” + HOR ABD with F of the elbow	In a sitting position with the arms elevated at 90° F of the shoulders in the sagittal plane with 90° F of the elbows and the hands facing the head, the patient was asked to do the “V scapular” and then extend the elastic band into horizontal abduction just until before the band touches the forehead. This exercise started with isometric work and progressed to isotonic work.
Exercise 12 Forward Punch “V scapular” in SP	In a sitting position with the shoulder in neutral, elbows flexed 90°, and an elastic band around the patient’s back in line with the patient’s forearms, the patient was asked to do the “V scapular” and then extend the elastic band forward in the scapular plane. This exercise started with isometric work and progressed to isotonic work.

Abbreviations: ABD, Abduction; F, Flexion; EXT ROT, External Rotation; HOR ABD, Horizontal Abduction, SP Scapular Plane.

**Table 4:** Progression guidelines [7].

Progression Guidelines:	
Exercise complexity	Two possible sources: (i) Mechanical load, which included exercise variations that required greater arm elevation angles or the use of weights; (ii) Task or motor planning–control difficulty, which involved tasks and exercises in which it is necessary to incorporate both feedforward and feedback mechanisms of motor performance [38,39].
Feedback from the EMGBF	Provided during all sessions to facilitate the best performance at each step. However, to progress to the next exercise or phase, the patient had to demonstrate their capability to reproduce the same performance without visual feedback. At this stage, EMGBF was used by the clinician to confirm the correct exercise performance.
Perceived effort	Although high-perceived effort is acceptable at the beginning of each phase or while increasing exercise complexity, correct exercise performance should be achieved with low perceived effort, pain-free exercise performance, and normal breathing.
Sets, repetitions and endurance	In the absence of normative data for endurance, exercises for this population were progressed when the patient could perform 3 sets of 10 repetitions or hold the specified position for 1 set of 10 repetitions of 10 s with no pain, low perceived effort (although high-perceived effort is acceptable at the beginning of each phase or while increasing exercise complexity), normal breathing, and good SSNC. Note: while these arbitrary performance criteria were effective for this population, the number of sets, repetitions, or holding time goal for progression will vary with different patient groups according to sport, work, and lifestyle requirements.
Resting time between exercises	Although patients were encouraged to rest the least time possible between exercises, they could rest for a maximum of 2 min between exercises (especially high-loaded exercises), but not between sets or repetitions [40].

**Table 5:** Resume of the testing procedure [7].

Outcome		Goal	Instrument	MCID	Assessment Procedures
Pain and Function		Determine pain intensity between assessment moments, measure and monitor function and symptoms over time	SPADI [43]	Ranging from 8 to 13 points [44]	Filling in the SPADI questionnaire
			NPRS [45]	2.17 [46]	Patient asked to report the worst pain felt in the last week
			DASH [47]	10.2 [44]	Filling in the DASH questionnaire
Scapular neuromuscular activity and control	SSNC	Assess the muscular percentage of MVIC activity of LT, SA and UT during arm elevation and lowering	EMGBF, Physioplux^TM^ system version 1.06	N/A	Raise then lower the arm, at a controlled self-paced velocity through maximum painless ROM, in the sagittal, scapular, and frontal planes from natural standing position for 1 set of 3 repetitions, with a 20 s pause between repetitions
	SSAO	Assess muscular activation onset during rapid active shoulder elevation	EMGBF, Physioplux^TM^ system version 1.06	N/A	Raise the arm, as rapidly as possible, without exacerbating pain or discomfort, to a maximum arm elevation angle of 45°, in the sagittal, scapular, and frontal planes from natural standing position for 1 set of 3 repetitions, with a 20 s pause between repetitions.
	Dynamic Scapular Alignment	Detect scapular dyskinesis	Clinical Observation of the scapular medial and inferior border [12]	N/A	Clinical Observation of the scapular medial and inferior border behavior during the arm elevation and lowering.
ROM		Assess glenohumeral ROM	Standard Goniometer [41]	N/A	Normative ROM assessment with a standard goniometer.
GMS		Assess glenohumeral flexor and abductor muscle strength	Isometric manual muscle testing [48]	N/A	Measured in a sitting position with the arm at 90° in the sagittal and frontal planes, respectively. Manual resistance was applied against the forearm with the elbow extended.

Abbreviations: MCID, Minimal Clinically Important Difference; SPADI, shoulder pain and disability index; NPRS, numeric pain rating scale; DASH, disabilities of the arm, shoulder, and hand; SSNC, scapular stabilizer neuromuscular control; MVIC, maximum voluntary isometric contraction; LT, lower trapezius; SA, serratus anterior; UT, upper trapezius; EMGBF, electromyographic biofeedback; ROM, range of motion; SSAO, scapular stabilizer activation onset; GMS, glenohumeral flexor and abductor muscle strength; N/A, non-applicable.

**Table 6:** Comparison of primary outcome pain and function within groups and between groups.

P_G	Initial	6 Weeks	95% CI	*t*-Test *p*^a^	Mean Change Values						
SPADI (0–100)	38.64 ± 11.61	3.32 ± 4.07	30.49 to 40.15	0.000	35.32 ± 10.33						
NPRS (0–10)	4.60 ± 1.39	0.50 ± 0.76	3.70 to 4.50	0.000	4.10 ± 0.85						
DASH (0–100 point)	35.60 ± 16.12	2.73 ± 3.25	25.77 to 39.97	0.000	32.87 ± 15.16						
**P+EMGBF_G**	**Initial**	**6 weeks**	**95% CI**	***t*-Test** ***p*** **^a^**	**Mean Change Values**	***t*-Test** **(Initial)** ***p*** **^b^**	***t*-Test** **(Final)** ***p*** **^b^**	***t*-Test** **(variation)** ***p*** **^b^**			
SPADI (0–100)	35.89 ± 12.20	3.02 ± 3.95	27.95 to 37.79	0.000	32.87 ± 10.50	0.470	0.814	0.462			
NPRS (0–10)	4.70 ± 1.30	0.40 ± 0.68	3.90 to 4.71	0.000	4.30 ± 0.86	0.816	0.664	0.466			
DASH (0–100 point)	35.60 ± 16.12	2.73 ± 3.25	24.24 to 35.62	0.000	29.93 ± 12.15	0.512	0.862	0.503			
**CT_G**	**Initial**	**6 weeks**	**95% CI**	***t*-Test** ***p*** **^a^**	**Mean Change Values**	***t*-Test** **(Initial)** ***p*** **^c^**	***t*-Test** **(Final)** ***p*** **^c^**	***t*-Test** **(variation)** ***p*** **^c^**	***t*-Test** **(Initial)** ***p*** **^d^**	***t*-Test** **(Final)** ***p*** **^d^**	***t*-Test** **(Variation)** ***p*** **^d^**
SPADI (0–100)	41.54 ± 18.11	11.02 ± 14.21	25.27 to 35.76	0.000	30.52 ± 11.20	0.551	0.025 *	0.167	0.255	0.020 *	0.497
NPRS (0–10)	4.40 ± 1.57	1.90 ± 1.12	3.08 to 3.92	0.000	2.50 ± 0.89	0.672	0.023 *	0.036 *	0.275	0.025 *	0.006 **
DASH (0–100 point)	38.31 ± 16.65	10.06 ± 10.96	23.64 to 32.87	0.000	28.26 ± 9.87	0.603	0.007 **	0.261	0.233	0.006 **	0.635

NOTE. Values are mean ± SD. ^a^ Within group. ^b^ Between groups P_G vs. P+EMGBF_G. ^c^ Between groups P_G vs. CT_G. ^d^ Between groups P+EMGBF_G vs. CT_G. Abbreviations: SD, standard deviation; CI, confidence interval; SPADI, shoulder pain and disability index; NPRS, numeric pain rating scale; DASH, daily life activities questions; * *p* < 0.05, ** *p* < 0.01 by paired *t*-test.

**Table 7:** Comparison of secondary outcomes within groups and between.

**P_G**		**Initial**	**6 Weeks**	**McNemar** ***p*** **^a^**							
SSNC	Diminished (reduced or moderate) (0–30% MVIC of LT and SA and <20% MVIC of UT)	16(80.00)	8(40.00)	0.008 **							
	Good (>30% MVIC of LT and SA and <20% MVIC of UT)	4(20.00)	12(60.00)								
SSAO (ms)	Feedback ^§^	8(40.00)	2(10.00)	0.031 *							
	Feedforward ^†^	12(60.00)	18(90.00)								
Dynamic Scapular Alignment	‘YES’ scapula dyskinesis (IB, MB, or both prominences)	20(100.00)	8(40.00)	0.000 ***							
	‘NO’ scapula dyskinesis (no prominences)	0(0.00)	12(60.00)								
ROM	Decreased	14(70.00)	3(15.00)	0.001 **							
	Normal (when the values corresponded with the normative ROM values expected for each movement and age group) [41] ^5^	6(30.00)	17(85.00)								
GMS	Decreased	16(80.00)	3(15.00)	0.000 ***							
	Normal (graded 5 when the patient withstood the test position against a strong pressure [48], for 3 s, without losing the testing position)	4(20.00)	17(85.00)								
**P+EMGBF_G**		**Initial**	**6 weeks**	**McNemar** ***p*** **^a^**	***p*** **^b^**	***p*** **^b^**	***p*** **^b^**				
SSNC	Diminished (reduced or moderate) (0–30% MVIC of LT and SA and <20% MVIC of UT)	15(75.00)	2(10.00)	0.000 ***	0.113	0.705	0.028 *				
	Good (>30% MVIC of LT and SA and <20% MVIC of UT)	5(25.00)	18(90.00)								
SSAO (ms)	Feedback ^§^	9(45.00)	1(5.00)	0.005 **	0.500 *	0.749	0.548				
	Feedforward ^†^	11(55.00)	19(95.00)								
Dynamic Scapular Alignment	‘YES’ scapula dyskinesis (IB, MB, or both prominences)	17(85.00)	4(20.00)	0.000 ***	0.744	0.072	0.168				
	‘NO’ scapula dyskinesis (no prominences)	3(15.00)	16(80.00)								
ROM	Decreased	12(60.00)	3(15.00)	0.012 *	0.592	0.507	1.000				
	Normal (when the values corresponded with the normative ROM values expected for each movement and age group) [41]	8(40.00)	17(85.00)								
GMS	Decreased	16(80.00)	4(20.00)	0.000 ***	0.744	1.000	0.677				
	Normal (graded 5 when the patient withstood the test position against a strong pressure [48], for 3 s, without losing the testing position)	4(20.00)	16(80.00)								
**CT_G**		**Initial**	**6 weeks**	**McNemar** ***p*** **^a^**	**X^2^** **(Initial)** ***p*** **^c^**	**X^2^** **(Final)** ***p*** **^c^**	**X^2^** **(variation)** ***p*** **^c^**	**McNemar** ***p*** **^a^**	**X^2^** **(Initial)** ***p*** **^d^**	**X^2^** **(Final)** ***p*** **^d^**	**X^2^** **(variation)** ***p*** **^d^**
SSNC	Diminished (reduced or moderate) (0–30% MVIC of LT and SA and <20% MVIC of UT)	17(85.00)	13(65.00)	0.125	0.168	0.677	0.133	0.000 ***	0.004 **	0.429	0.000 ***
	Good (>30% MVIC of LT and SA and <20% MVIC of UT)	3(15.00)	7(35.00)								
SSAO (ms)	Feedback ^§^	9(45.00)	3(15.00)	0.031 *	1.000	0.749	0.663	0.008 *	0.507	1.000	0.292
	Feedforward ^†^	11(55.00)	17(85.00)								
Dynamic Scapular Alignment	‘YES’ scapula dyskinesis (IB, MB, or both prominences)	17(85.00)	14(70.00)	0.250	0.003 **	0.072	0.057	0.000 *	0.001 **	1.000	0.001 **
	‘NO’ scapula dyskinesis (no prominences)	3(15.00)	6(30.00)								
ROM	Decreased	14(70.00)	3(15.00)	0.002 **	0.752	0.736	1.000	0.012 **	0.591	0.744	1.000
	Normal (when the values corresponded with the normative ROM values expected for each movement and age group) [41]	6(30.00)	17(85.00)								
GMS	Decreased	16(80.00)	5(25.00)	0.001 **	0.519	1.000	0.429	0.000 ***	0.749	1.000	0.705
	Normal (graded 5 when the patient withstood the test position against a strong pressure [48], for 3 s, without losing the testing position)	4(20.00)	15(75.00)								

NOTE. Values are mean ± SD. ^a^ Within group. ^b^ Between groups P_G vs. P+EMGBF_G. ^c^ Between groups P_G vs. CT_G. ^d^ Between groups P+EMGBF_G vs. CT_G. ^†^ The feedforward activation onset represents the anticipatory muscle activation that occurs prior to the mobilizer muscles, and the feedback activation onset is a muscle activation that occurs after the designated feedforward period [49]. By definition, and used for this study, a feedforward activation pattern (considered as normal) was the activation of the lower trapezius (LT) and serratus anterior (SA) 100 ms before to 50 ms after the anterior deltoid (AD) activation onset [49]. ^§^ A feedback pattern was an activation of the LT and SA greater than 50 ms after AD activation [49]. Abbreviations: SD, standard deviation; X^2^, chi-square test; SSNC, scapular stabilizer neuromuscular control; SSAO, scapular stabilizer activation onset; IB, inferior border of the scapula; MB, medial border of the scapula; ROM, range of motion; GMS, glenohumeral flexors and abductors isometric muscle strength; * *p* < 0.05, ** *p* < 0.01, *** *p* < 0.001 by chi-square test.

## Data Availability

The datasets generated and/or analyzed during the current study contain personal health information and are not publicly available due to privacy and ethical restrictions. De-identified data and analysis code may be available from the corresponding author on reasonable request, subject to approval by the Ethics Committee of the Alcoitão Superior School of Health and a data-use agreement.

## Abbreviations

The following abbreviations are used in this manuscript:

ABD	Abduction
CI	Confidence Interval
cm	Centimeters
CT_G	Control Therapy Group
DASH	Disabilities of the arm, shoulder, and hand
EMG	Electromyography
EMGBF	Electromyographic Biofeedback
EXT ROT	External Rotation
F	Flexion
GMS	Glenohumeral Flexor and Abductor Muscle Strength
HOR ABD	Horizontal Abduction
IB	Inferior Border of the Scapula
Kg	Kilogram
LT	Lower Trapezius
MB	Medial Border of the Scapula
MCID	Minimal Clinically Important Difference
mm	Millimeters
ms	Milliseconds
MVIC	Maximum Voluntary Isometric Contractions
N/A	Non-Applicable
NPRS	Numeric Pain Rating Scale
P+EMGBF_G	Scapular-Focused Exercise Protocol + EMGBF group
P_G	Protocol Group
RCS	Rotator Cuff-Related Pain Syndrome
ROM	Range of Motion
SA	Serratus Anterior
SD/sd	Standard Deviation
SP	Scapular Plane
SPADI	Shoulder Pain and Disability Index
SPSS	Statistical Package for the Social Sciences
SSNC	Scapular Stabilizer Neuromuscular Control
SSAO	Scapular Stabilizer Activation Onset
USA	United States of America
UT	Upper Trapezius
X^2^	Chi-Square Test
